# Alternative splicing and nonsense-mediated decay regulate telomerase reverse transcriptase (TERT) expression during virus-induced lymphomagenesis *in vivo*

**DOI:** 10.1186/1471-2407-10-571

**Published:** 2010-10-21

**Authors:** Souheila Amor, Sylvie Remy, Ginette Dambrine, Yves Le Vern, Denis Rasschaert, Sylvie Laurent

**Affiliations:** 1 Equipe TLVI, Université François Rabelais de Tours, UFR Sciences et Techniques, Parc de Grandmont 37200 Tours France; 2 INRA-Département de Santé Animale - Centre de recherches de Tours - 37380 Nouzilly - France; 3 Centre de recherches INRA de Tours-UR IASP - Service de Cytométrie - 37380 Nouzilly-France

## Abstract

**Background:**

Telomerase activation, a critical step in cell immortalization and oncogenesis, is partly regulated by alternative splicing. In this study, we aimed to use the Marek's disease virus (MDV) T-cell lymphoma model to evaluate TERT regulation by splicing during lymphomagenesis *in vivo*, from the start point to tumor establishment.

**Results:**

We first screened cDNA libraries from the chicken MDV lymphoma-derived MSB-1 T- cell line, which we compared with B (DT40) and hepatocyte (LMH) cell lines. The chTERT splicing pattern was cell line-specific, despite similar high levels of telomerase activity. We identified 27 alternative transcripts of chicken TERT (chTERT). Five were in-frame alternative transcripts without *in vitro *telomerase activity in the presence of viral or chicken telomerase RNA (vTR or chTR), unlike the full-length transcript. Nineteen of the 22 transcripts with a premature termination codon (PTC) harbored a PTC more than 50 nucleotides upstream from the 3' splice junction, and were therefore predicted targets for nonsense-mediated decay (NMD). The major PTC-containing alternatively spliced form identified in MSB1 (ie10) was targeted to the NMD pathway, as demonstrated by UPF1 silencing. We then studied three splicing events separately, and the balance between in-frame alternative splice variants (d5f and d10f) plus the NMD target i10ec and constitutively spliced chTERT transcripts during lymphomagenesis induced by MDV indicated that basal telomerase activity in normal T cells was associated with a high proportion of in-frame non functional isoforms and a low proportion of constitutively spliced chTERT. Telomerase upregulation depended on an increase in active constitutively spliced chTERT levels and coincided with a switch in alternative splicing from an in-frame variant to NMD-targeted variants.

**Conclusions:**

TERT regulation by splicing plays a key role in telomerase upregulation during lymphomagenesis, through the sophisticated control of constitutive and alternative splicing. Using the MDV T-cell lymphoma model, we identified a chTERT splice variant as a new NMD target.

## Background

The telomeric enzyme complex, consisting of a telomerase reverse transcriptase (TERT) and an RNA template (TR), adds terminal telomeric repeats to the end of the chromosome, to maintain telomere length during cell proliferation [1,2]. Most normal somatic cells lack telomerase activity, whereas telomerase activation is observed in proliferating cells and cancer cells [3,4]. Telomerase activity is highly regulated in lymphocytes, being expressed only in activated lymphocytes [5]. In humans, the hTR transcript is constitutively produced, whereas the production of hTERT is highly regulated at both the transcriptional and post-transcriptional levels [6,7]. Alternative splicing of the hTERT transcript plays a role in this regulation and 10 alternatively spliced sites have been identified in the hTERT gene [8]. Notably, the alpha isoform, corresponding to an in-frame deletion in the RT motif, appears to be a dominant inhibitor of telomerase activity [9,10].

Recent studies have demonstrated that alternative splicing is an important gene regulation mechanism, helping to increase the diversity of proteins by favoring the production of large numbers of isoforms with dominant positive or negative functions [11]. However, almost one third of all alternative transcripts harbor a premature termination codon (PTC) and most are thought to be degraded by the nonsense-mediated mRNA decay (NMD) pathway [12].

NMD occurs in all eukaryotic cells. This cell surveillance system detects and rapidly degrades aberrant mRNAs containing PTCs (for reviews, see [13,14]). Termination codons are generally considered premature if they occur more than 50 to 55 nucleotides upstream from a final splice site recognized by the exon junction complex (EJC). The EJC serves as a platform for the binding of UPF factors, which are considered to be the conserved core of the NMD machinery. UPF1 is an ATP-dependent RNA helicase and RNA-dependent ATPase activated by phosphorylation. It is a crucial element of the NMD machinery, because silencing of the *upf1 *gene results in the stabilization of PTC-containing mRNAs in all organisms in which NMD has been investigated. This surveillance mechanism not only eliminates abnormal transcripts, but also controls transcript levels through a global system known as RUST (regulated unproductive splicing and translation) [15,16]. Several recent studies have demonstrated that the role of alternative splicing in gene regulation has been largely underestimated and have shown that this process is involved in homeostatic regulation, pathogenesis [17] and, particularly, in cancers [18,19].

Gallid herpesvirus 2 (GaHV-2), also known as Marek's Disease Virus (MDV), is an avian oncogenic alphaherpesvirus that induces T-cell lymphomas in chickens. These tumors develop within six weeks of infection in birds, making this model a unique asset for studies of the kinetics of disease induction and progression in a natural host system. This model has already proved valuable for studies of human lymphoma [20]. Indeed, Marek's disease is the only naturally occurring model for human lymphomas involving the overexpression of CD30 [21]. GaHV-2 is also the only virus harboring a viral homolog of the telomerase RNA template (vTR), with a sequence 88% identical to that of the chicken telomerase RNA (chTR). Functional analysis has shown that vTR can reconstitute telomerase activity by interacting with chicken TERT (chTERT) more efficiently than chTR [22]. Furthermore, vTR expression increases during GaHV-2 lymphomagenesis and this increase is correlated with the upregulation of telomerase activity, which is not associated with an upregulation of chTERT transcription [23,24]. Telomerase is also activated by a number of human oncogenic viruses [25], but investigations in this case are obviously restricted to *in vitro *analyses of cell lines derived from tumors or transformed *in vitro*. The chicken telomerase has a number of key features in common with the human enzyme, in terms of the organization of the TERT and TR genes and the regulation of telomerase activity [26]. Furthermore, as for hTERT, many alternative variants of chTERT have been identified and are thought to play a role in the regulation of telomerase activity [26,27]

In this study, we investigated the alternative splicing of chTERT in the MDV lymphomagenesis model. We report the identification of 27 alternative transcripts of chicken TERT cloned during a comparison of three different chicken cell lines including MSB-1, a MDV tumor cell line. Five alternative transcripts were in-frame transcripts, whereas the other 22 were found to harbor a PTC. We then demonstrated that the in-frame transcripts generated proteins with no telomerase activity *in vitro*, whereas telomerase activity was detected for the products of constitutively spliced transcripts. The major PTC-containing variant found in the MDV cell line was shown to be a target of NMD in an upf1 silencing test. Finally, investigation of the splicing-mediated regulation of chTERT *in vivo*, in MDV-infected chickens, demonstrated that basal telomerase activity in normal T cells was associated with a high ratio of in-frame non-functional isoforms to functional constitutively spliced chTERT. During the upregulation of telomerase associated with lymphomagenesis, an increase in active constitutively spliced chTERT transcript levels was observed that coincided with a switch in alternative splicing from the in-frame type to the NMD type. These results suggest that the regulation of telomerase activity is partly dependent on the fine regulation of splicing of chTERT, regulating the abundance of functional chTERT mRNA.

## Results

### Complex profile of chTERT alternative transcripts

**Figure 1:**
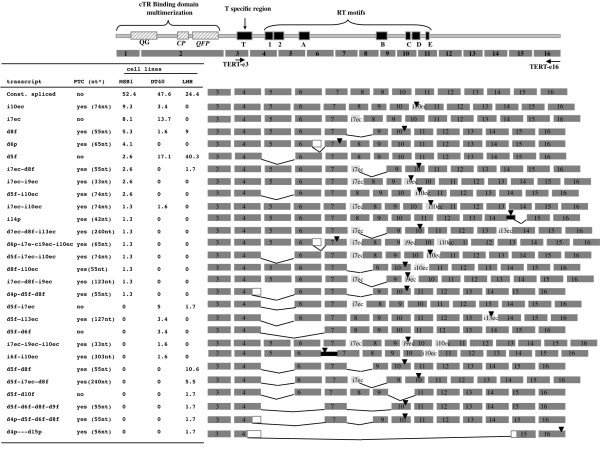
**Alternative transcripts of chTERT identified in three avian cell line**. At the top, we show a schematic diagram of telomerase protein, showing major conserved protein motifs, including reverse transcriptase domains 1 and 2 and the A, B, C, D and E motifs, and of the chTERT gene, with its 16 exons shown as gray boxes. The PCR primers are shown as arrows below the diagram. Each alternative transcript is shown on the right, with the splicing event depicted as a clear gray box for insertion of the exon cassette; dark lines indicate deletion, black boxes indicate intron retention and white boxes indicate the deletion of part of an exon. Positions of premature stop codons (PTCs) are indicated by black triangles. On the left, we show the name of the spliced transcripts, the presence or absence of a PTC with its position relative to the 3' exon-exon junction and represented as a function of cell line. The name of the transcript is indicated on the left and is coded as follow: iXec for insertion of exon cassette X, dXf for full deletion of exon X, dXp for partial deletion of exon X, iXp for retention of part of intron X and iXf for insertion of full intron X.

We first compared the pattern of alternative chTERT transcript production from the MDV T- MSB-1 cell line with that of two other cell lines: the DT40 B cell line, and the epithelial LMH cell line. Three cDNA libraries encompassing the T and RT regions of chTERT were generated (Figure 1).

The ratio of constitutively to alternatively spliced TERT transcripts was the first major difference identified between cell lines. Constitutively spliced transcripts accounted for about half of all transcripts in both lymphoid cell lines (52.4% and 47.6% for MSB1 and DT40, respectively), but only 24.4% of transcripts in the LMH cell line. However, no significant differences in telomerase activity were observed between the three cell lines (telomerase activity of 152272 for MSB1, 165818 for DT40 and 151115 for LMH) and there was therefore no clear correlation between this ratio and telomerase activity. In addition to constitutively spliced chTERT, we identified 27 alternative transcripts generated through the five typical splicing events: exon skipping, inclusion of a new exon cassette, cases of intron retention, use of alternative splice donor sites and use of alternative splice acceptor sites. Most of the alternative transcripts were generated by one of the four frequent splicing events: i7ec, i10ec, d5f and d8f. Each cell line displayed a number of rare specific transcripts generated by a specific combination of the four major events or a combination of one or more of these events with a rare splicing event (Figure 1).

The LMH cell line displayed a preferential deletion of exons, (Figure 1). By contrast, lymphoid cell lines were characterized by the insertion of introns generated by exonization (inclusion of intronic sequences in constitutive transcripts), through the formation of exon cassettes or the retention of all or part of an intron (Figure 1). Nevertheless, we observed differences in the distribution of splicing events between MSB1 T cells and DT40 B cells. For instance, major differences were seen for one of the major splicing events, i10ec (alone or in association with other splicing events), which was more frequently detected in MSB-1 than in DT40 cells (28.3% *versus *8.2%).

**Figure 2:**
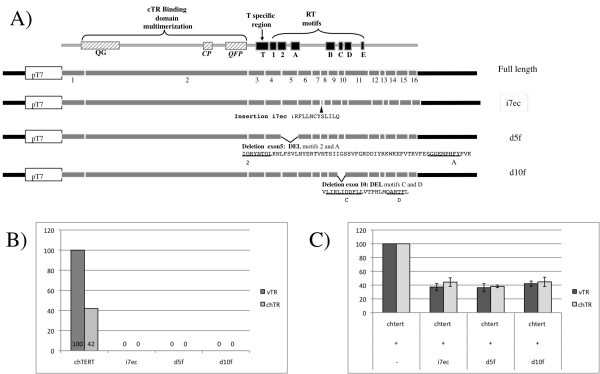
**chTERT in-frame isoforms are non functional in the *in vitro *telomerase assay**. (A) Schematic diagram of the 3 reconstituted in-frame isoforms of chTERT inserted into pcDNA under the control of the T7 promoter. Amino acids deleted or inserted in each isoform are indicated and those belonging to the RT motif are underlined. Plasmids were used to produce recombinant *in vitro*-translated proteins that were incubated in the presence of *in vitro*-transcribed vTR or chTERT before the TRAP assay. (B) The histogram shows telomerase activity relative to the value obtained with vTR in the presence of the full-length constitutively spliced chTERT transcript arbitrary set at 100%. (C) Histograms show telomerase activities for a mixture (1:1) of *in vitro*-translated full-length chTERT and the indicated isoform in the presence of vTR or chTR. The values obtained with the mixture of full-length chTERT and isoform are expressed relative to the value for the full-length chTERT, arbitrarily set at 100%. The histogram shows the mean and standard deviation obtained from 3 biological analyses.

Most of the detected alternative transcripts (22/27) harbored premature termination codons (PTCs) (Figure 1). Nineteen of these 22 transcripts harbored a PTC located more than 50 nucleotides upstream from the 3' splice junction and were predicted to result in NMD-sensitive transcripts. Of the five remaining transcripts encoding in-frame isoforms of chTERT, only the i7ec transcript, corresponding to an inclusion of 12 amino acids (aa) between regions A and B of the protein, preserved the catalytic site of TERT, the four remaining transcripts involving the deletion of exon 5 (d5f), resulting in the deletion of amino acids from motifs 2 and A (Figure 1 and 2).

### None of the in-frame isoforms of chTERT is functional

We assessed the telomerase activity of three reconstituted in-frame isoforms (Figure 2). Isoforms i7ec and d5f were identified as alternative transcripts (Figure 1) and isoform d10f was reconstructed for individual testing of the effect of the d10f splicing event, on which we subsequently focused specifically. We inserted the cDNAs of the isoforms downstream from the T7 promoter, for the assessment of telomerase activity in an *in vitro *assay [28]. Telomerase activity was measured in the presence of *in vitro *transcribed vTR or chTR. Consistent with published results [28], the full-length chTERT transcript reconstituted an efficient telomerase complex with both TRs with vTR that was 58% more efficient than chTR, whereas none of the in-frame spliced isoforms reconstituted telomerase activity. Thus, i7ec, despite the preservation of its RT motifs, displayed no telomerase activity and, as expected, in-frame isoforms with deletions in the RT domains were not able to reconstitute telomerase activity. As exon 5 (d5f) was deleted from all the four remaining in-frame isoforms other than i7ec, (Figure 1), we can conclude that all the in-frame isoforms are inactive. We then assessed the dominant negative function of these isoforms on telomerase activity when produced together with the active full-length chTERT (Figure 2C). All the isoforms acted as negative regulators, with inhibition levels of 74% for d5f to 58% for d10f when compared with the exclusive expression of full-length chTERT with vTR. No significant difference was seen between chTR and vTR, demonstrating that the inhibition mechanism did not depend on the nucleotide differences identified between vTR and chTR [28]. Thus, all the chTERT isoforms tested seemed to have dominant negative effects on telomerase activity, whether with chTR or vTR.

### The i10ec variant undergoes NMD

**Figure 3:**
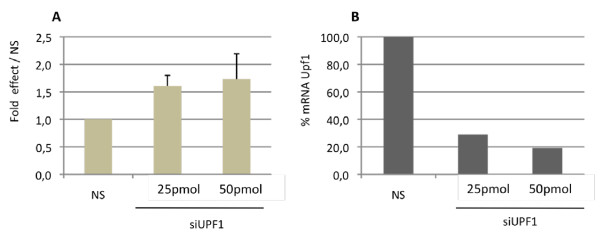
**Downregulation of the i10ce variant by NMD**. (A) Upf1 depletion increases levels of the chTERT splice variant i10ce. Levels of i10ce are expressed relative to total chTERT levels. Peak areas of i10ec were obtained by capillary electrophoresis analysis of the PCR products targeting exon 10, generated from cDNA from 1 well of P6 transfected with 25 pmol or 50 pmol of siUPF-1. The histogram shows the mean and standard deviation obtained from 4 biological analyses. All values are expressed as a fold difference with respect to "no silencing" (NS), for which the value was fixed at 1. (B) siUpf1 knockdown of Upf1. The histogram shows Upf1 mRNA levels, calculated by RT-PCR analysis. Upf1 levels in each sample were normalized with respect to GAPDH levels for the corresponding sample. The values obtained for "no silencing" (NS) LMH cells were set at 100%.

The i10ec transcript was the most frequently represented transcript with predicted sensitivity to NMD in the MSB1 cell line. We therefore followed this transcript during lymphomagenesis. We evaluated its sensitivity to NMD by blocking the NMD pathway, using siRNA to target UPF1, the major NMD factor in the transfected LMH cell line. We chose to study LMH cells for technical reasons, essentially linked to transfection efficiency. Indeed MSB1 (or DT40) cells are lymphocytes and are difficult to transfect (2 to 5% transfection efficiency associated with high mortality rates of 70-90%). As i10ec was not detected in chTERT alternative transcripts of LMH (Figure 1), we first determined whether transcripts harboring the i10ec splice event and thus defined as variant i10ec were detectable by PCR on LMH cDNA followed by fragment capillary electrophoresis analysis assays (CEAA) (see additional file 1: comparison of cDNA library and fragment analysis). This more sensitive technique, focusing on the specific amplification, by PCR, of short fragments, led to detection of the i10ec variant at a frequency of 1 to 5% with respect to full-length chTERT, thus rendering silencing experiments feasible. We observed significant increases in relative i10ec levels of 1.6 and 1.7 when the NMD pathway was inhibited with 25 and 50 pmol of siRNA against UPF1, respectively, as shown by comparisons with a non-silenced sample used as a control and normalized to 1 (Figure 3A). The effective targeting of UPF1 by siRNA was also confirmed by monitoring UPF1 mRNA levels. UPF1 was strongly downregulated by 25 and 50 pmol of siRNA UPF1 (71 and 81%, respectively) (Figure 3B). These results demonstrate that the i10ec variant was efficiently detected and degraded by NMD.

### The increase in telomerase activity during the lymphomagenesis induced by MDV is related to the upregulation of constitutively spliced chTERT at the expense of alternatively spliced in-frame isoforms

Finally, we investigated the contribution of chTERT alternative splicing to the regulation of telomerase activity during the course of the lymphomagenesis induced by MDV. As it is difficult to amplify cDNA from the relatively long chTERT mRNA fragment present at low abundance in PBLs, we developed two independent fragment capillary electrophoresis analysis assays for investigating the dynamics of chTERT splicing regulation *in vivo*. In our assay, we chose to monitor changes in the ratio of the constitutively spliced transcript to variants resulting from two major splicing events, d5f and i10ec, identified in the MSB1 cell line, currently used as the reference cell line in investigations of Marek's disease lymphoma and displaying some of the characteristics of lymphoma cells *in vivo*. Variant d5f encodes a non-functional isoform of chTERT and i10ec, generating a variant targeted by the NMD pathway. Furthermore, the assay detecting the i10ec variant also detected the d10f variant, which would also be expected to generate a non-functional isoform, as demonstrated by the *in vitro *telomerase assay (Figure 2). Our assays were first optimized with the CD4+-sorted MSB1 cell line. The results obtained in fragment capillary electrophoresis analysis assays of cDNA samples were similar to those obtained for library analysis, although the ratios differed very slightly (see additional file 1: comparison of cDNA library and fragment analysis). These results confirm that fragment analysis is an appropriate technique for studying spliced chTERT and is suitable for use with sorted chicken CD4^+ ^T cells.

**Figure 4:**
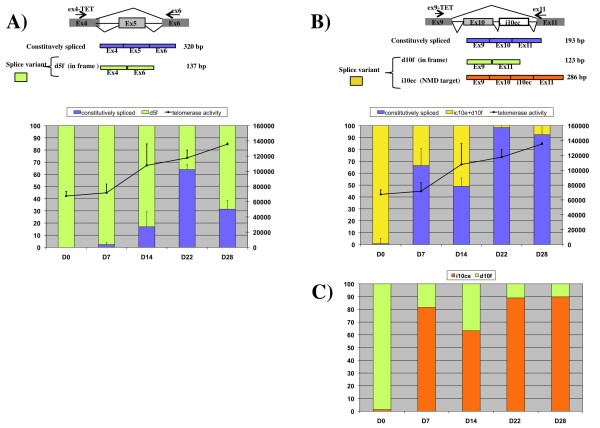
**Regulation of chTERT splicing involving variants 5 and 10 during lymphomagenesis *in vivo***. Proportions of constitutively spliced chTERT transcript (in blue) and alternatively spliced variants d10f+i10ec (in yellow) and the d5f variant (in green), as shown in the panels on the right and left, respectively. The proportions correspond to the peak area obtained by capillary electrophoresis analysis of PCR targeting exon 5 (A) or 10 (B), performed on cDNA extracted from sorted CD4^+ ^T cells sampled from GaHV-2-infected chickens at 5 different time points after infection, as indicated on the x-axis. The curve shows telomerase activity, which was obtained by summing the peak areas corresponding to the elongation products (right y-axis) (C) Proportions of d10f (green) and i10ec (orange) variants, as indicated above the graph and corresponding to 100% of alternative splice variants10 at each time point after infection, as shown in (B).

We first measured telomerase activity at five previously identified crucial time points (d0, d7, d14, d22 and d28) [24](Figure 4). The results obtained with CD4^+ ^T cells were similar to those obtained with PBL: (i) basal telomerase activity was detected in CD4^+ ^T cells from non infected chickens (67527), (ii) a slight induction of telomerase activity was first observed at 7 days (71750), corresponding to the phase of primary semi-productive infection (iii), this induction peaked at 14 days p.i. (107800), corresponding to the expected time of lymphoma onset and (iv) persisted until 28 days p.i. (135310). Capillary electrophoresis analysis of splicing variants 5 and 10 was then performed on cDNA extracted from the same CD4^+ ^T-cell samples. Variants 5 and 10 had three major features in common: i) almost all the chTERT transcripts detected in CD4+ T cells from non infected chickens in tests for variants 5 and 10 were alternatively spliced (99.8% and 99.3% respectively), ii) in detection tests for both these variants, constitutively spliced transcripts became detectable from day 7 p.i., and their detection coincided with the increase in telomerase activity, iii) constitutively spliced transcript levels increased throughout infection (Figure 4).

However, the changes in splicing pattern differed between variants 5 and 10. For the d5f variant, we observed a progressive increase in the levels of constitutively spliced transcripts from day 7 (2.3%), peaking at d22 (63.9%) (Figure 4A). By contrast, with variant 10 (encompassing both d10f and i10ec), the levels of constitutively spliced transcript increased considerably between d0 and d7 (from 0.7% to 66.4%) and remained high until day 28 (92.4%) (Figure 4B). Investigation of the ratio of d10f to i10ec showed a clear shift from d10f to i10ec at d7 (Figure 4C). Thus, at d0, the d10f variant, was, with d5f, almost the only alternatively spliced variant detectable. Then, beginning on d7, the proportion of the i10ec variant, which was predicted to be sensitive to NMD, increased strongly (>60%). These results indicate that (i) variants encoding in-frame non functional isoforms (d5f and d10f) predominate in uninfected CD4^+ ^T cells and are associated with basal levels of telomerase activity (ii) the activation of telomerase is accompanied by an increase in the proportion of constitutively spliced transcripts and a decrease in the proportion of in-frame alternatively spliced transcripts, favoring the predicted NMD-sensitive transcript.

## Discussion

The regulation of telomerase activity is a complex process involving several steps operating at both the transcriptional and post-transcriptional levels. In recent years, TERT splicing has been extensively studied in many types of human cells, tissues and tumors and in other organisms, such as plants, nematodes and ciliates [29]. A number of different alternative transcripts have been described, but little is known about the cause of splicing variant production and the ways in which splicing variant levels are regulated [29]. This is particularly true for the regulation of splicing during the cell transformation process and the dynamics of this process *in vivo*. In this study, we carried out RT-PCR fragment analysis to study the dynamics of the ratio of the constitutively spliced productive form of chTERT to three alternatively spliced non productive forms -- one NMD-sensitive transcript and two in-frame transcripts -- in chicken CD4^+ ^T cells, during a lymphomagenic process induced by MDV, an oncogenic herpes virus, in its natural host. In our model, lymphoma was observed on day 28 p.i. in 100% of infected chickens, facilitating analyses of the dynamics of gene regulation during oncogenesis *in vivo *[24]. Another major and unique advantage of our model is that we were able to control the start of tumorigenesis through the inoculation of chickens with the virus. Using this approach, we have previously shown that telomerase activation begins seven days after infection and peaks 22 days after infection, this time point being correlated with the first detection of the tumor in animals. Furthermore, telomerase activation and lymphomagenesis were correlated with upregulation of the vTR transcript, but no upregulation of the chTR and chTERT transcripts was seen [23,24]. In this study, we investigated telomerase regulation in more detail, by following the regulation of chTERT splicing in this model. Constitutively spliced transcript was almost undetectable at d0 in CD4^+ ^T lymphocytes, but its levels gradually increased, peaking 22 days after infection, on the day of lymphoma onset. These data are the first to be obtained *in vivo *and are consistent with previous *in vitro *observations after the *v-Rel *transformation of spleen cells from chickens [27]. Moreover, the regulation of telomerase activity in human T lymphocytes seems to be similar, as full-length hTERT is undetectable in normal T cells and an increase in telomerase activity in activated T cells is associated with the induction of full-length hTERT mRNA production [30]. We also confirmed the results obtained for *in vitro *analyses of human tissues, with telomerase activation involving the induction, or a switch to production of the full-length hTERT mRNA, as shown in various tissues [31-34].

Studies on spliced hTERT transcripts have generally focused on two major isoforms of hTERT: alpha (in-frame isoform with a 36 bp deletion in motif A) and beta (isoform introducing a PTC in exon 10) [35]. The in-frame alpha isoform of hTERT has been shown to regulate telomerase activity negatively, acting as a dominant negative inhibitor [9,10]. We identified five in-frame isoforms of chTERT, all of which had modifications affecting the RT motifs of telomerase. We have shown that three reconstituted in-frame isoforms, i7ec, d5f and d10f, are inactive, being unable to reconstitute telomerase activity *in vitro *with either vTR or chTR. These isoforms also seem to act as negative regulators, as the association of the full-length chTERT transcript with an in-frame isoform decreased telomerase activity by a factor of 2 to 2.5 in the *in vitro *telomerase assay (Figure 2C). We can therefore hypothesize that the low basal levels of telomerase activity in normal T lymphocytes are controlled by in-frame non-functional isoforms, as these are almost the only isoforms detected (>99%). The basal telomerase activity observed at d0 (Figure 4) was associated with very low levels of constitutively spliced active chTERT transcripts (0.5%), consistent with a previous study [36] demonstrating that telomerase-positive cell lines contain only a few molecules of functional hTERT mRNA.

We also observed that telomerase activation associated with an increase in constitutively spliced transcript levels was accompanied by a switch in the profile of alternative transcripts from in-frame transcripts encoding non functional isoforms to NMD-sensitive transcripts. The ratio of the NMD-sensitive i10ec to the in-frame d10f increased markedly with the upregulation of telomerase activity, from d7 onwards (Figure 4). The PTC of i10ec was located 74 nucleotides upstream from the last exon-exon junction, consistent with the definition of PTCs targeted by the NMD pathway. Nevertheless, the NMD sensitivity of the PTC-containing i10ec transcript was definitively confirmed by UPF1 silencing, which increased i10ec levels by a factor of up to 1.7 (Figure 3), consistent with previous reports of a increase in mRNA levels by a factor of 1.8 for PTC-containing COL alpha-2 [37] or H-ras [38]. The chTERT i10ec splice variant was thus identified as a new NMD target. These results confirm the hypothesis put forward by Chang H and Delany M. E. (2006) in a study identifying 19 chTERT spliced variants in different cell types, 16 of which (including i10ec) were predicted to have a PTC leading to degradation by the NMD pathway.

**Figure 5:**
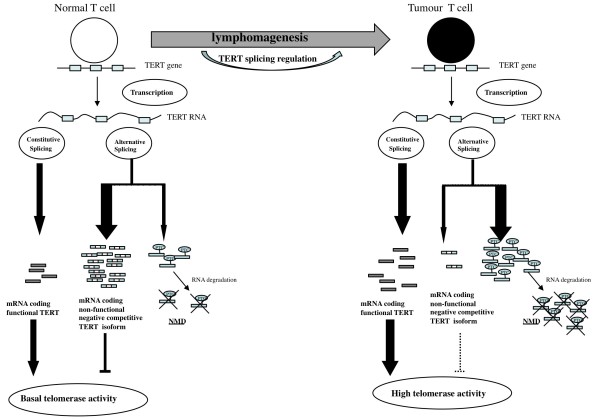
**Model of the splicing regulation of chTERT during lymphomagenesis**.

The change in the ratio of d10f to i10ec strongly suggests that differential splicing in favor of the NMD-sensitive transcript may be regulated by splicing activators or repressors, leading to the overrepresentation of constitutively spliced transcripts with respect to in-frame negative regulator isoforms. Recent studies have indicated that NMD is a sophisticated tool in physiological autoregulatory gene expression [12]. Lewis *et al*. [15] suggested that the coupling of splicing and NMD results in RUST, the mechanism regulating the ratio of productive and unproductive spliced forms of many genes, as demonstrated for the splicing factor SC35 [39]. Figure 5 schematically represents a model accounting for our observations. In lymphocytes, in which the ratio of constitutively spliced and in-frame negative regulator isoforms of TERT must be tightly controlled to maintain basal telomerase activity, the ratio of NMD-sensitive transcripts may be a major sensor (Figure 5). Moreover, with telomerase upregulation, the switch to NMD-sensitive transcripts may contribute to downregulation of the in-frame isoform acting as a negative regulator (Figure 5). Global regulation of this type, involving both an in-frame isoform and an NMD-sensitive transcript, has been described for serum response factor (SRF) [40]. In this system, the abundance of SRF is reduced by RUST, but SRF gene expression is also regulated by an in-frame isoform of SRF repressing transcription of the SRF gene itself. In our model, this feedback mechanism not only makes use of the active transcript ratio, but is also based on the negative regulator ratio. Interestingly, this model has also been proposed for regulation of the abundance of SR protein [41].

## Conclusion

Our findings strongly suggest that the regulation of TERT splicing plays a key role in the upregulation of telomerase activity *in vivo*, by controlling the proportion of active constitutively spliced transcripts with an in-frame negative regulator and a third partner, an NMD-sensitive transcript. Regulation of the ratio of NMD-sensitive transcript to in-frame transcript encoding the negative regulator isoform also plays a key role in suppressing telomerase activity in normal cells, in which negative regulator isoforms predominate, whereas NMD-sensitive transcripts become predominant when telomerase is upregulated during oncogenesis.

## Methods

### Cell line culture and experimental assay *in vivo*

Three avian cell lines were used: GaHV-2 lymphoma-derived MSB-1 T cells [42] currently used as the reference cell line in Marek's disease investigation, the B-cell line DT40 [43] and the LMH hepatocellular carcinoma cell line [44]. Six-week-old White Leghorn specified pathogen-free susceptible B13B13 chickens were infected with GaHV-2 RB-1B virus [45]. Blood was obtained from two birds at 7, 14 and 22 days post-infection (dpi) and from one bird at 28 dpi, as the other bird had already died. The first time point (0 dpi), corresponded to the pooling of 2 ml blood samples from each of the chickens, taken before infection. All experimental procedures were conducted in accordance with approved protocols for the use of animals in research.

### Transient transfection with siRNA

Transient transfection with siRNA was carried out in six-well plates. LMH cells were transfected with 25 or 50 pmol of siRNA per well, in the presence of Lipofectamine 2000 reagent (Invitrogen), according to the manufacturer's protocol. Cells were harvested 48 hours later for RT-PCR analysis of UPF1, GAPDH and chTERT splicing variant i10ec. The siRNA targeting UPF1 (siUPF1) [GGUGGAAGAUGUAAUUAUU] was designed and purchased from Eurogentec.

### Flow cytometry analysis and cell sorting

Peripheral blood lymphocytes (PBL) were isolated from blood [46] and immunolabeled for the sorting of CD4^+ ^T cells. The CD4 antigen was detected by incubation with the monoclonal antibody (Ab) CD4-UNLB, 8210-01 (Southern Biotechnology), followed by goat anti-mouse Ig conjugated to R-phycoerythrin (PE) (Jackson Immunoresearch). The cells were then analyzed by flow cytometry and PE-labeled cells were sorted and recovered for RNA and protein extraction, with a MoFlo™ apparatus (Beckmann Coulter, Fort Collins, USA) and a high-speed cell sorter. The sorting speed was around 35,000 cells per second.

### RNA extraction, cDNA synthesis and PCR amplification

Total RNA was extracted from 2 × 10^6 ^cells, with the RNAble Kit (Eurobio). The cDNA was synthesized with SuperScript III reverse transcriptase (Invitrogen), using oligo (dT) priming (Eurogentec), according to the manufacturer's instructions. The RT region of the *chTERT *gene, and *gapdh *and *upf1 *genes were amplified by PCR in Ready Master Mix 1.1X (Abgene), using three pairs of primers: TERT-e3 with TERT-e16 (Table 1, Figure 1A), GAPDHs (GTCCTCTCTGGCAAAGTCCAAG) with GAPDHr (CCACAACATACTCAGCACCTGC) designed from the *Gallus gallus gapdh *sequence (NCBI genebank number M11213.1) and UPF1s (GGGCTCCGAGTTCGAGTTCA) with UPF1r (CAGAATGCCATCCGGTCCAT) from the predicted sequence of the *Gallus gallus upf1 *gene (NCBI reference sequence NC_006115.2). The PCR products for UPF1 and GAPDH were analyzed by agarose gel electrophoresis. Adobe Photoshop 7.0 software (Adobe Systems, USA) was used to calculate the signal intensities for UPF1 relative to the GAPDH loading control.

**Table 1:** Primers used in the study

**Oligonucleotide**^**1**^	**Localization**^**2**^	**Sequence**^**3**^	Screening
tert e3-M331s	e3 (2210-39)	CTGTTCCTGCCTATGAACATTGTTACCGTG	chTERT cDNA
tert e3M137-s	e3 (2260-80)	CTATACTGGCTGATGGATTCC	exon 4
M139s	e4 (2480-500)	CATCAAGGCTCCGGTTCATTC	exon 5
M141s	e5 (2680-700)	GGGAGAGATGATATCTACAGG	exon 6
M142r	e5 (2700-680)	CCTGTAGATATCATCTCTCCC	exon 4
M143s	e6 (2815-35)	GTGGAAGTGATATCACAGGTC	exon 7
M144r	e6 (2835-15)	GACCTGTGATATCACTTCCAC	exon 5
M145s	e7 (2974-92)	GTGTCCAAGCTTCAAGAGA	exon 8
M146r	e7 (2992-74)	TCTCTTGAAGCTTGGACAC	exon 6
M147s	e8 (3040-60)	AATGAGAACAGTTCCACCCTG	exon 9
M148r	e8 (3060-40)	CAGGGTGGAACTGTTCTCATT	exon 7
M149s	e9 (3172-92)	AGCTTATGCTACGGAGACATG	exon 10
M150r	e9 (3192-72)	CATGTCTCCGTAGCATAAGCT	exon 8
M151s	e10 (3258-79)	GCTGGTTACGCCACATTTAATG	exon 11
M152r	e10 (3279-58)	CATTAAATGTGGCGTAACCAGC	exon 9
M153s	e11 (3373-93)	GATGATATCCCGGGATGTTCC	exon 12
M154r	e11 (3393-73)	GGAACATCCCGGGATATCATC	exon 10
M155s	e12 (3556-76)	TGCAAATTGACTGCAGTCCTC	exon 13
rM156r	e12 (3576-56)	GAGGACTGCAGTCAATTTGCA	exon 11
M157s	e13 (3618-42)	CAGCCTTCAGACAGTTCTAATTAAC	exon 14
M158r	e13 (3642-18)	GTTAATTAGAACTGTCTGAAGGCT	exon 12
M159s	e14 (3727-47)	CCTGATTTCTTCCTAAGGATC	exon 15
M160r	e14 (3747-27)	GATCCTTAGGAAGAAATCAGG	exon 13
M162r	e15 (3870-50)	ATGGTAGCACAGCCATTCTGC	exon 14
M163r	e16 (3996-76)	CACCGTCTTCAGCAGTTCCAT	exon 15
tert e16-M316-r	e16 (4041-07)	TTAGTCCAGTATAGTTTTGAAATCTTGACAAAGCG	chTERT cDNA
TET-tert e4	e4 (2554-78)	TET-CAGAAACTCAGCAAGGAAAGCAGAG	splice variant 5
tert e6	e6 (2874-55)	CCACCTTATTCCATAGACAG	splice variant 5
TET-tert e9	e9 (3170-92)	TET-GCAGCTTATGCTACGGAGACATG	splice variant 10
tert e11	e11 (3361-42)	TCACCACAGTCTTCTTGGCA	splice variant 10

^1 ^name of the primer with TET labeling indication,

^2 ^localization in the chTERT cDNA sequence AY626231 (NCBI n°)

^3 ^written in the 5'->3' orientation

### Generation and screening of cell-line cDNA libraries

PCR products corresponding to the chTERT RT region were inserted into the pGEM-T Easy vector (Promega) and positive clones were randomly selected after screening for the presence of the insert by PCR. Individual selected clones were then screened for the type of alternative transcript, by running PCR with a panel of primer pairs (Table 1) covering each of the exons (4 to 15) of the chTERT cDNA. For the validation of exon PCR screening, 13 representative clones were also completely sequenced (MWG Biotech). Sequences were analyzed with DNASTAR sequence analysis software (Lasergene).

### PCR fragment analysis of chTERT splicing variants 5 and 10

The ratios of constitutively to alternatively spliced transcripts from cell lines and sorted CD4^+ ^T cells were determined by fluorescent fragment analysis [23,24]. Nested-PCR was performed on the first PCR amplification product of the chTERT RT region, in Ready Master Mix 1.1X (Abgene), with specific tetrachlorofluorescein phosphoramidite-labeled forward primers and unlabeled reverse primers specific for exon 5 or 10 (Table 1). Amplification products were analyzed with an automated ABI Prism 310 fragment analyzer (Perkin Elmer Life Sciences) and the ratio of spliced transcripts was determined as a percentage of the peak area for constitutively (C) versus alternatively (A) spliced transcripts. Each assay was performed at least three times.

### Reconstitution *in vitro *of the telomerase complex

The three cDNAs for alternative transcripts of chTERT -- d5f, d10f, and i10ec -- were inserted into pcDNA 3 and the telomerase complex was reconstituted [28]. We incubated 1 μg of vTR or chTR RNA transcribed *in vitro *with T7 polymerase, and 2 μl of alternatively spliced and full-length chTERT transcripts expressed in the TNT system (Promega). We assessed the inhibition of telomerase activity by the isoforms with 2 μl of the *in vitro*-translated full-length isoform and 2 μl of *in vitro*-translated isoform. Aliquots of ribonucleoprotein assembly products were used for the assessment of telomerase activity in the telomere repeat amplification protocol (TRAP) assay [28].

### Telomere repeat amplification protocol (TRAP) assay

The telomerase activity of sorted CD4^+ ^T cells from chicken in *in vivo *infection assays and of *in vitro *reconstituted complexes was quantified as previously described [28,24]. Briefly, telomerase activity was quantified with 500 ng of protein extracted from CD4+ T cells [24] or 0.3 μl of *in vitro *ribonucleoprotein assembly products [28]. PCR was carried out with the 6-carboxytetramethylrhodamine-labeled forward primer TS (5'-AATCCGTGCAGCAGAGTT-3') and CX-ext (5'-CCCTAACCCTAACCCTAACCCTAA-3') as the reverse primer. An internal amplification standard (ITAS) was added to the PCR mixture in quantitative TRAP assays or was used independently as a PCR control. PCR products were analyzed by capillary electrophoresis (ABI Prism 310; PerKinElmer Life Sciences). The telomerase activity of each protein extract was estimated by adding together the integrated values for each telomerase elongation product of at least 60 bp.

## Abbreviations

TERT: telomerase reverse transcriptase; TR: telomerase RNA; NMD: nonsense-mediated decay; PTC: premature termination codon, TRAP: telomere repeat amplification protocol

## Competing interests

The authors declare that they have no competing interests.

## Authors' contributions

Study concept and design: SL and DR; Acquisition of data SA, SR, YLV, GD and SL; Analysis and interpretation of data: SA, SL, GD and DR; Drafting of manuscript: SA, SL; Critical revision of manuscript SL, GD and DR: Obtainment of funding: SL, GD and DR; Study supervision SL, DR. All authors have read and approved the final manuscript.

## Pre-publication history

The pre-publication history for this paper can be accessed here:

http://www.biomedcentral.com/1471-2407/10/571/prepub

## Supplementary Material

Additional file 1 **Comparison of estimates of the levels of splicing variants 5 and 10 by cDNA cloning and fragment electrophoresis**. Proportions of constitutively spliced chTERT transcript (in blue) and alternatively spliced variants d10f (in green), 10ec (in orange) and d5f (in green). The proportions correspond to the peak area obtained by capillary electrophoresis analysis (CEAA) of PCR targeting variant 10 (A) or 5 (B), performed on cDNA extracted from LMH or MSB1 cells (3 biological analyses) or obtained from cDNA analysis by estimation of the percentage of all alternative transcripts harboring splicing event 5 or 10 (Figure 1). Click here for file

## References

[B1] Greider CW Blackburn EH Identification of a specific telomere terminal transferase activity in *Tetrahymena *extracts Cell 1985 43 2 Pt 1 405 413 10.1016/0092-8674(85)90170-9 3907856

[B2] Dong CK Masutomi K Hahn WC Telomerase: regulation, function and transformation Crit Rev Oncol Hematol 2005 54 2 85 93 10.1016/j.critrevonc.2004.12.005 15843091

[B3] Kim NW Piatyszek MA Prowse KR Harley CB West MD Ho PL Coviello GM Wright WE Weinrich SL Shay JW Specific association of human telomerase activity with immortal cells and cancer Science 1994 266 5193 2011 2015 10.1126/science.7605428 7605428

[B4] Janknecht R On the road to immortality: hTERT upregulation in cancer cells FEBS Lett 2004 564 1-2 9 13 10.1016/S0014-5793(04)00356-4 15094035

[B5] Pan C Xue BH Ellis TM Peace DJ Diaz MO Changes in telomerase activity and telomere length during human T lymphocyte senescence Exp Cell Res 1997 231 2 346 353 10.1006/excr.1997.3475 9087176

[B6] Feng J Funk WD Wang SS Weinrich SL Avilion AA Chiu CP Adams RR Chang E Allsopp RC Yu J The RNA component of human telomerase Science 1995 269 5228 1236 1241 10.1126/science.7544491 7544491

[B7] Cong YS Wright WE Shay JW Human telomerase and its regulation Microbiol Mol Biol Rev 2002 66 3 407 425 table of contents 10.1128/MMBR.66.3.407-425.2002 12208997 PMC120798

[B8] Saeboe-Larssen S Fossberg E Gaudernack G Characterization of novel alternative splicing sites in human telomerase reverse transcriptase (hTERT): analysis of expression and mutual correlation in mRNA isoforms from normal and tumour tissues BMC Mol Biol 2006 7 26 10.1186/1471-2199-7-26 16939641 PMC1560392

[B9] Yi X White DM Aisner DL Baur JA Wright WE Shay JW An alternate splicing variant of the human telomerase catalytic subunit inhibits telomerase activity Neoplasia 2000 2 5 433 440 10.1038/sj.neo.7900113 11191110 PMC1507981

[B10] Colgin LM Wilkinson C Englezou A Kilian A Robinson MO Reddel RR The hTERTalpha splice variant is a dominant negative inhibitor of telomerase activity Neoplasia 2000 2 5 426 432 10.1038/sj.neo.7900112 11191109 PMC1507985

[B11] Kim E Magen A Ast G Different levels of alternative splicing among eukaryotes Nucleic Acids Res 2007 35 1 125 131 10.1093/nar/gkl924 17158149 PMC1802581

[B12] Muhlemann O Eberle AB Stalder L Zamudio Orozco R Recognition and elimination of nonsense mRNA Biochim Biophys Acta 2008 1779 9 538 549 18657639 10.1016/j.bbagrm.2008.06.012

[B13] Rehwinkel J Raes J Izaurralde E Nonsense-mediated mRNA decay: Target genes and functional diversification of effectors Trends Biochem Sci 2006 31 11 639 646 10.1016/j.tibs.2006.09.005 17010613

[B14] Silva AL Romao L The mammalian nonsense-mediated mRNA decay pathway: to decay or not to decay! Which players make the decision? FEBS Lett 2009 583 3 499 505 10.1016/j.febslet.2008.12.058 19162024

[B15] Lewis BP Green RE Brenner SE Evidence for the widespread coupling of alternative splicing and nonsense-mediated mRNA decay in humans Proc Natl Acad Sci USA 2003 100 1 189 192 10.1073/pnas.0136770100 12502788 PMC140922

[B16] Lareau LF Brooks AN Soergel DA Meng Q Brenner SE The coupling of alternative splicing and nonsense-mediated mRNA decay Adv Exp Med Biol 2007 623 190 211 full_text 18380348 10.1007/978-0-387-77374-2_12

[B17] Nissim-Rafinia M Kerem B The splicing machinery is a genetic modifier of disease severity Trends Genet 2005 21 9 480 483 10.1016/j.tig.2005.07.005 16039004

[B18] Kim E Goren A Ast G Insights into the connection between cancer and alternative splicing Trends Genet 2008 24 1 7 10 10.1016/j.tig.2007.10.001 18054115

[B19] Pettigrew CA Brown MA Pre-mRNA splicing aberrations and cancer Front Biosci 2008 13 1090 1105 10.2741/2747 17981615

[B20] Osterrieder N Kamil JP Schumacher D Tischer BK Trapp S Marek's disease virus: from miasma to model Nat Rev Microbiol 2006 4 4 283 294 10.1038/nrmicro1382 16541136

[B21] Burgess SC Young JR Baaten BJ Hunt L Ross LN Parcells MS Kumar PM Tregaskes CA Lee LF Davison TF Marek's disease is a natural model for lymphomas overexpressing Hodgkin's disease antigen (CD30) Proc Natl Acad Sci USA 2004 101 38 13879 13884 10.1073/pnas.0305789101 15356338 PMC518847

[B22] Fragnet L Blasco MA Klapper W Rasschaert D The RNA subunit of telomerase is encoded by Marek's disease virus J Virol 2003 77 10 5985 5996 10.1128/JVI.77.10.5985-5996.2003 12719590 PMC154048

[B23] Shkreli M Dambrine G Soubieux D Kut E Rasschaert D Involvement of the oncoprotein c-Myc in viral telomerase RNA gene regulation during Marek's disease virus-induced lymphomagenesis J Virol 2007 81 9 4848 4857 10.1128/JVI.02530-06 17314164 PMC1900149

[B24] Debba-Pavard M Ait-Lounis A Soubieux D Rasschaert D Dambrine G Vaccination against Marek's disease reduces telomerase activity and viral gene transcription in peripheral blood leukocytes from challenged chickens Vaccine 2008 26 38 4904 4912 10.1016/j.vaccine.2008.07.038 18680776

[B25] Bellon M Nicot C Regulation of telomerase and telomeres: human tumor viruses take control J Natl Cancer Inst 2008 100 2 98 108 10.1093/jnci/djm269 18182620

[B26] Chang H Delany ME Complicated RNA splicing of chicken telomerase reverse transcriptase revealed by profiling cells both positive and negative for telomerase activity Gene 2006 379 33 39 10.1016/j.gene.2006.04.021 16806743

[B27] Hrdlickova R Nehyba J Liss AS Bose HR Mechanism of telomerase activation by v-Rel and its contribution to transformation J Virol 2006 80 1 281 295 10.1128/JVI.80.1.281-295.2006 16352553 PMC1317554

[B28] Fragnet L Kut E Rasschaert D Comparative functional study of the viral telomerase RNA based on natural mutations J Biol Chem 2005 280 25 23502 23515 10.1074/jbc.M501163200 15811851

[B29] Sykorova E Fajkus J Structure-function relationships in telomerase genes Biol Cell 2009 101 7 375 392 371 p following 392 10.1042/BC20080205 19419346

[B30] Jalink M Ge Z Liu C Bjorkholm M Gruber A Xu D Human normal T lymphocytes and lymphoid cell lines do express alternative splicing variants of human telomerase reverse transcriptase (hTERT) mRNA Biochem Biophys Res Commun 2007 353 4 999 1003 10.1016/j.bbrc.2006.12.149 17204238

[B31] Fan Y Liu Z Fang X Ge Z Ge N Jia Y Sun P Lou F Bjorkholm M Gruber A Differential expression of full-length telomerase reverse transcriptase mRNA and telomerase activity between normal and malignant renal tissues Clin Cancer Res 2005 11 12 4331 4337 10.1158/1078-0432.CCR-05-0099 15958614

[B32] Villa R Porta CD Folini M Daidone MG Zaffaroni N Possible regulation of telomerase activity by transcription and alternative splicing of telomerase reverse transcriptase in human melanoma J Invest Dermatol 2001 116 6 867 873 10.1046/j.1523-1747.2001.01343.x 11407973

[B33] Lincz LF Mudge LM Scorgie FE Sakoff JA Hamilton CS Seldon M Quantification of hTERT splice variants in melanoma by SYBR green real-time polymerase chain reaction indicates a negative regulatory role for the beta deletion variant Neoplasia 2008 10 10 1131 1137 18813352 10.1593/neo.08644 PMC2546589

[B34] Yokoyama Y Wan X Takahashi Y Shinohara A Tamaya T Alternatively spliced variant deleting exons 7 and 8 of the human telomerase reverse transcriptase gene is dominantly expressed in the uterus Mol Hum Reprod 2001 7 9 853 857 10.1093/molehr/7.9.853 11517292

[B35] Kilian A Bowtell DD Abud HE Hime GR Venter DJ Keese PK Duncan EL Reddel RR Jefferson RA Isolation of a candidate human telomerase catalytic subunit gene, which reveals complex splicing patterns in different cell types Hum Mol Genet 1997 6 12 2011 2019 10.1093/hmg/6.12.2011 9328464

[B36] Yi X Shay JW Wright WE Quantitation of telomerase components and hTERT mRNA splicing patterns in immortal human cells Nucleic Acids Res 2001 29 23 4818 4825 10.1093/nar/29.23.4818 11726691 PMC96692

[B37] Usuki F Yamashita A Kashima I Higuchi I Osame M Ohno S Specific inhibition of nonsense-mediated mRNA decay components, SMG-1 or Upf1, rescues the phenotype of Ullrich disease fibroblasts Mol Ther 2006 14 3 351 360 10.1016/j.ymthe.2006.04.011 16807116

[B38] Barbier J Dutertre M Bittencourt D Sanchez G Gratadou L de la Grange P Auboeuf D Regulation of H-ras splice variant expression by cross talk between the p53 and nonsense-mediated mRNA decay pathways Mol Cell Biol 2007 27 20 7315 7333 10.1128/MCB.00272-07 17709397 PMC2168895

[B39] Sureau A Gattoni R Dooghe Y Stevenin J Soret J SC35 autoregulates its expression by promoting splicing events that destabilize its mRNAs EMBO J 2001 20 7 1785 1796 10.1093/emboj/20.7.1785 11285241 PMC145484

[B40] Zhang X Azhar G Huang C Cui C Zhong Y Huck S Wei JY Alternative splicing and nonsense-mediated mRNA decay regulate gene expression of serum response factor Gene 2007 400 1-2 131 139 10.1016/j.gene.2007.06.008 17629633

[B41] Palusa SG Reddy AS Extensive coupling of alternative splicing of pre-mRNAs of serine/arginine (SR) genes with nonsense-mediated decay New Phytol 2010 185 1 83 89 10.1111/j.1469-8137.2009.03065.x 19863731

[B42] Akiyama Y Kato S Two cell lines from lymphomas of Marek's disease Biken J 1974 17 3 105 116 4616680

[B43] Baba TW Giroir BP Humphries EH Cell lines derived from avian lymphomas exhibit two distinct phenotypes Virology 1985 144 1 139 151 10.1016/0042-6822(85)90312-5 2998040

[B44] Kawaguchi T Nomura K Hirayama Y Kitagawa T Establishment and characterization of a chicken hepatocellular carcinoma cell line, LMH Cancer Res 1987 47 16 4460 4464 3607775

[B45] Djeraba A Bernardet N Dambrine G Quere P Nitric oxide inhibits Marek's disease virus replication but is not the single decisive factor in interferon-gamma-mediated viral inhibition Virology 2000 277 1 58 65 10.1006/viro.2000.0576 11062036

[B46] Djeraba-AitLounis A Soubieux D Klapper W Rasschaert D Induction of telomerase activity in avian lymphoblastoid cell line transformed by Marek's disease virus, MDCC-MSB1 Vet Pathol 2004 41 4 405 407 10.1354/vp.41-4-405 15232141

