# Supplementary figures and images for: Alternative splicing and nonsense-mediated decay regulate telomerase reverse transcriptase (TERT) expression during virus-induced lymphomagenesis in vivo
Source: BMC Cancer. 2010 Oct 21;10:571. doi: 10.1186/1471-2407-10-571 (PMC2976754; doi:10.1186/1471-2407-10-571)

## Slide 1
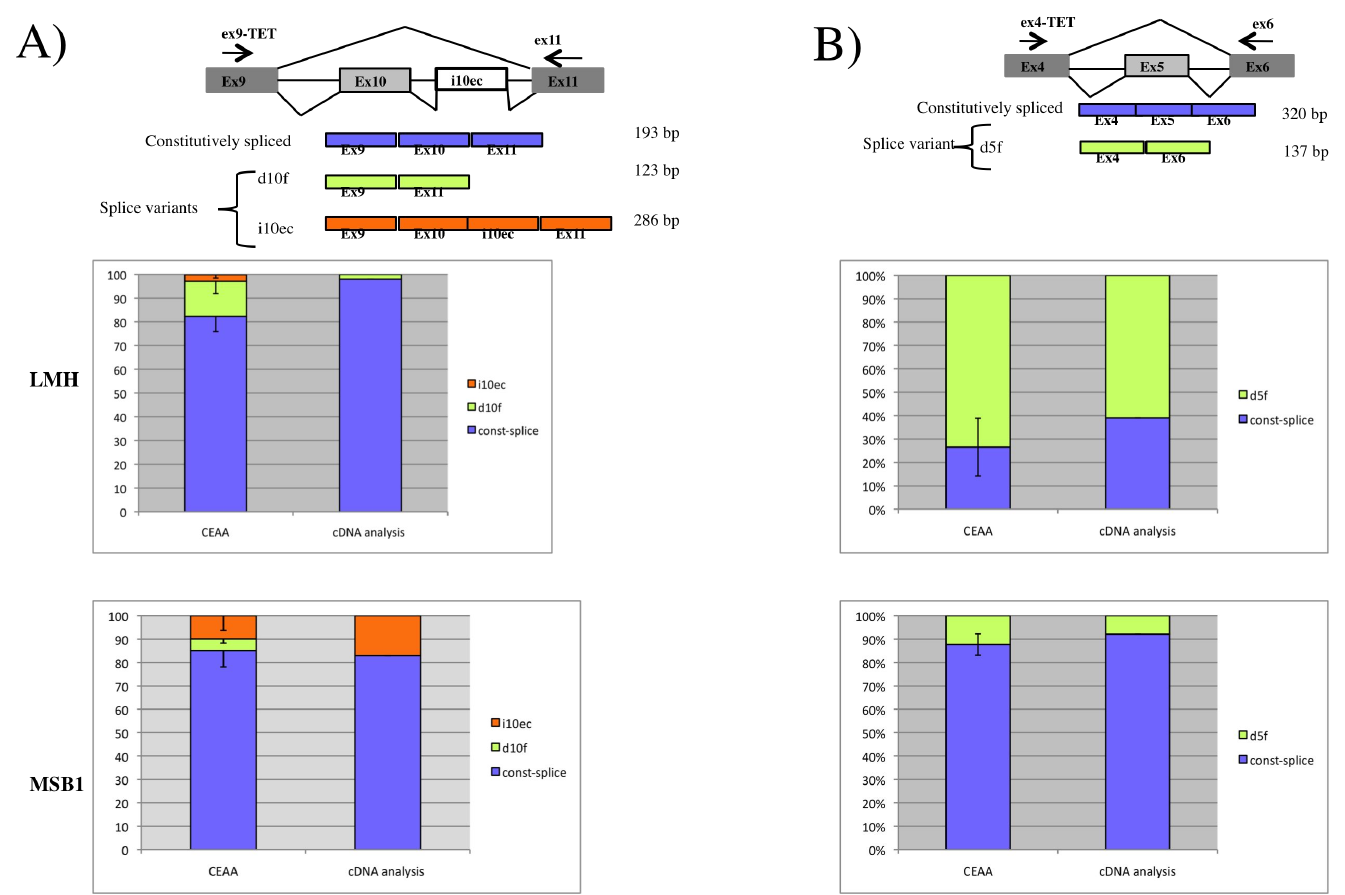

Supplement: Additional file 1 — Comparison of estimates of the levels of splicing variants 5 and 10 by cDNA cloning and fragment electrophoresis. Proportions of constitutively spliced chTERT transcript (in blue) and alternatively spliced variants d10f (in green), 10ec (in orange) and d5f (in green). The proportions correspond to the peak area obtained by capillary electrophoresis analysis (CEAA) of PCR targeting variant 10 (A) or 5 (B), performed on cDNA extracted from LMH or MSB1 cells (3 biological analyses) or obtained from cDNA analysis by estimation of the percentage of all alternative transcripts harboring splicing event 5 or 10 (Figure 1). [file 1471-2407-10-571-S1.PPT]
